# Alternatives in the treatment of prosthetic infection after the Bentall-de Bono operation.

**DOI:** 10.1186/1749-8090-10-S1-A361

**Published:** 2015-12-16

**Authors:** R MartinezSanz, R Ávalos, P Garrido, R de la Llana, JJ Jiménez, J Montoto, M Brouard, JL Iribarren, PC Prada, C VaqueroPuerta

**Affiliations:** 1 Complejo Hospitalario Universitario de Canarias, Tenerife, Spain; 2 Universidad de La Laguna, Tenerife, Spain; 3 Universidad de Valladolid, Valladolid, Spain

## Background

Infection of a valved conduit used to perform a Bentall-De Bono technique is an infrequent but serious complication.

## Objective

Two different strategies of treatment, based on the extension of the infection, were used. We report our experience on the management of three cases so treated.

## Method

In case 1, three months after hospital discharge, a small fistula in the upper sternal scar was observed. When it was explored surgically, the fistula affected the superior-posterior part of the sternum and the pericardium was covering the prosthesis except in a small area over the left distal anastomosis of the dacron graft, which had a drop of pus. Cases 2 and 3 presented clear mediastinitis, with fever, leucocytosis and purulent effusion around the conduit, at 7 and 10 days after Bentall procedure. In case 1 s. *Epidermidis *was isolated and in cases 2 and 3 enterococci were cultured.

## Results

In case 1, a pedicled left internal mammary artery flap was inserted among the Dacron and the pericardium but not prosthesis replacement was performed. The postoperative course was excellent and the patient is well six years later. In cases 2 and 3, the valved Dacron tube was removed. A homograft was put in place. In both cases there was no problem with the homograft. This worked well, but mediastinitis took several weeks to heal. Finally, both patients were discharged and continue well 5 and 2 years later.

## Conclusions

If a very mild infection by a not aggressive germ affects a valved conduit, a coverage of the Dacron with a pedicled mammary flap may be effective. Otherwise, putting a homograft is the solution.

